# Social isolation and cancer management – advanced rectal cancer with patient delay following the 2011 triple disaster in Fukushima, Japan: a case report

**DOI:** 10.1186/s13256-017-1306-3

**Published:** 2017-05-16

**Authors:** Akihiko Ozaki, Claire Leppold, Toyoaki Sawano, Masaharu Tsubokura, Manabu Tsukada, Tetsuya Tanimoto, Masahiro Kami, Hiromichi Ohira

**Affiliations:** 1 Department of Surgery, Minamisoma Municipal General Hospital, 2-54-6 Takamicho, Haramachi, Minamisoma, Fukushima 975-0033 Japan; 2 Department of Research, Minamisoma Municipal General Hospital, Minamisoma, Fukushima 975-0033 Japan; 3 Department of Radiation Protection, Minamisoma Municipal General Hospital, Minamisoma, Fukushima 975-0033 Japan; 4 Department of Internal Medicine, Jyoban Hospital of Tokiwa Foundation, Iwaki, Fukushima 972-8322 Japan; 5 Medical Governance Research Institute, Minato-ku, Tokyo 108-0074 Japan; 6 Global Public Health Unit, School of Social and Political Science, University of Edinburgh, EH8 9LD, Edinburgh, UK

**Keywords:** Patient delay, Colorectal cancer, Social isolation, Social support, Social change, Social relationships, Fukushima, Nuclear disaster, Health information, Elderly

## Abstract

**Background:**

Little is known about the effects of social isolation in the elderly on their process of gaining health information and seeking health care.

**Case presentation:**

In March 2011, Fukushima, Japan experienced an earthquake, tsunami, and nuclear disaster, also known as Japan’s triple disaster. In June 2016, an 80-year-old Japanese man, who lived alone after divorce at the age of 42, presented to our hospital with bloody stools and dizziness. Although his bloody stools initially occurred in May 2015, a year earlier, he did not pursue the possibility of malignancy. He was diagnosed as having stage IIIA rectal cancer. Detailed history taking revealed that he experienced social isolation after the disaster, due to the evacuation of his friends, losing his regular opportunities for socialization. He additionally reported that the current diagnosis of rectal cancer made him feel he had lost his health in addition to his social relationships. Although radical surgery was attempted, it failed to resect the lesion completely, and thereafter his disease gradually progressed. As support from family or friends was not available, he was not able to receive palliative radiation therapy or home-based care in his end-of-life period. He died at a long-term care facility in February 2017.

**Conclusions:**

This case suggests that intense social isolation after the Fukushima disaster was a likely contributor to the patient delay, poor treatment course, and poor outcome of an elderly patient with rectal cancer. Direct communication with family and friends may play an indispensable role in increasing health awareness and promoting health-seeking behaviors, and in the midst of social isolation, elderly patients with cancer may lose these opportunities and experience increased risk of patient delay. Although health care providers may be able to alleviate isolation-induced delay by promoting cancer knowledge and awareness widely among local residents, policy-led interventions at the community level may be essential to reducing social isolation and its health consequences.

## Background

Colorectal cancer is the third and second most common cancer among males and females worldwide, respectively [1]. High-income countries (HICs), including Japan, have recently achieved a steady decline in colorectal cancer mortality rates, primarily due to the start of national screening programs [2]. Nonetheless, not all populations have benefited from these programs. Less than half of targets for colorectal screening programs in HICs actually undergo recommended tests [3, 4], as exemplified in the case of Japan, where the attendance rate of annual fecal occult blood tests was only 35.4% in 2013 [4]. Resultantly, approximately 80% of cases are detected after symptoms appear in HICs [5], and 25.0 to 36.6% of patients with symptomatic colorectal cancer further experience patient delay, which is defined as 3 months or longer from symptom recognition to first medical consultation [6]. The limited uptake in screening programs and prominence of patient delay call for an improvement in public awareness of screenings, symptoms, and consequences of colorectal cancer [6, 7].

It has been suggested that social support may help the patients make timely medical consultations, although multiple elements can contribute to patient delay in patients with colorectal cancer, including poor health literacy, rural residence, and denial of symptoms or non-recognition of symptom seriousness [8]. However, the elderly may not be able to obtain sufficient social support; although this population tend to rely on family and friends to gather health information [9, 10], they may experience deterioration of social relationships due to deaths of significant others or physical distance from family members [11]. In fact, the prevalence of social isolation, absolute lack of social relationships, currently reaches 10 to 43% among the elderly in HICs, with evidence of its various health consequences [11]. Although the extent of social isolation among the elderly has not been well described in the nationwide scale of Japan, it has been shown that 17.4% and 38.4% of the population aged 65 years or older lived alone and only with their partners, respectively, in 2014 [12]. In addition, in Japan the proportion of the general population who never or rarely socialize with those other than family members was 15.3%, the highest figure among countries which belong to Organisation for Economic Co-operation and Development (OECD), from 1999 to 2002 [13]. Thus, we hypothesize that a large proportion of the elderly population may struggle to obtain the health information and social support necessary to remain healthy or recover from episodes of ill-health. However, limited information is available regarding how the processes of health information-seeking among the elderly may be affected by social isolation. Nuclear disasters are a phenomenon in which the affected area may experience a long-term alteration of its social structure, and can provide a unique lens to assess the ways in which social isolation may impact health information-seeking behaviors among the elderly.

**Fig. 1:**
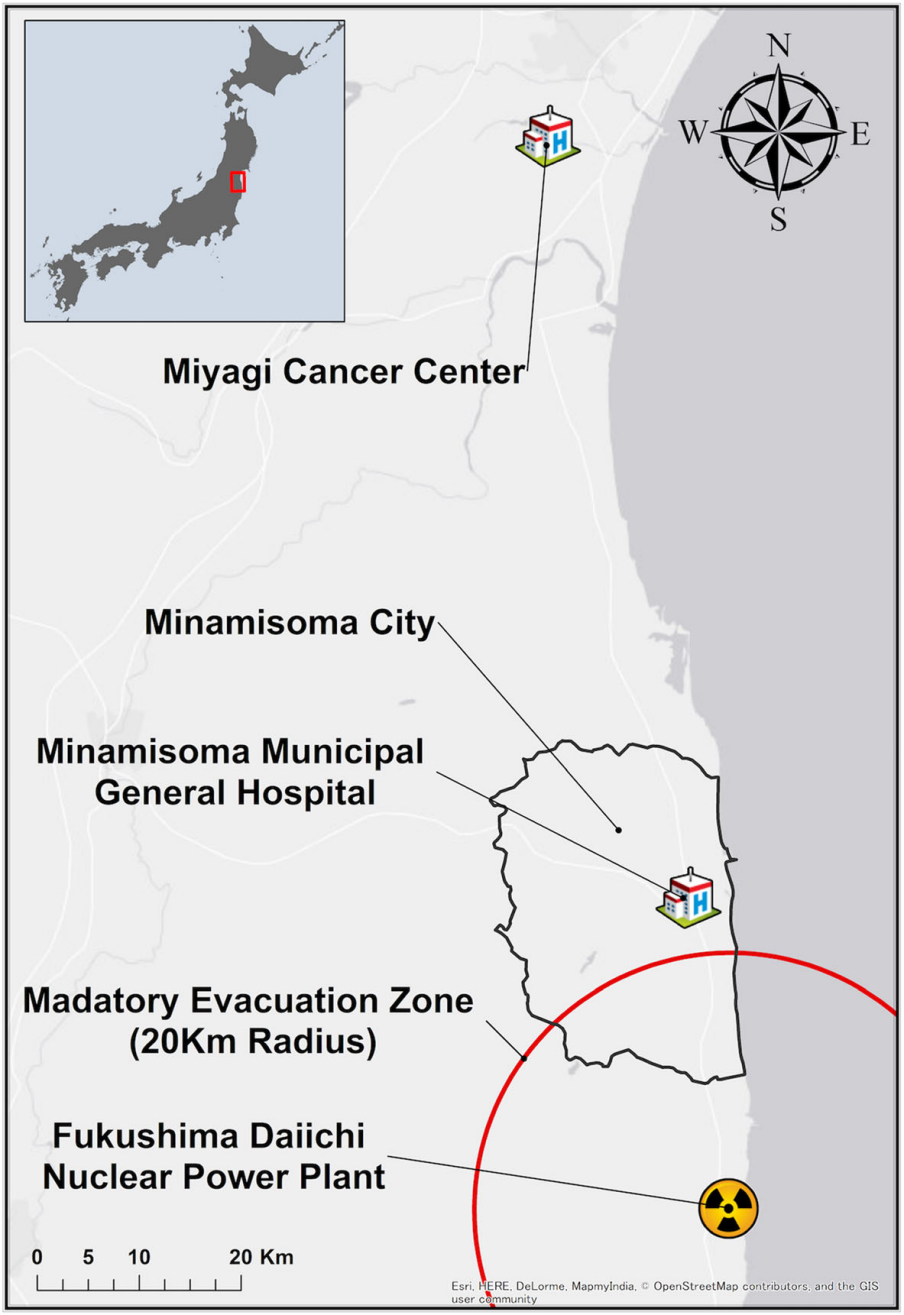
Geographical location of Minamisoma City, Minamisoma Municipal General Hospital, and Miyagi Cancer Center

In March 2011, Minamisoma City, Fukushima Prefecture (Fig. 1), located 14 to 38 km north of Fukushima Daiichi Nuclear Power Plant (FDNPP), was struck by all three components of Japan’s 2011 triple disaster (earthquake, tsunami, and nuclear disaster) [14]. A 20 km radius from the FDNPP was designated as a mandatory evacuation zone by the Central Government of Japan. Minamisoma City Office has continuously examined the level of external and internal radiation exposure among local residents, and has demonstrated that those figures are too low to cause health effects in the general public [15–18]. A recent study reported that there have been no clear relationships between the incidence of childhood thyroid cancer and the level of external radiation exposure in Fukushima Prefecture, denying the existence of a radiation-induced pandemic for this malignancy [19]. However, the city experienced long-term changes in its social structure, primarily due to mass evacuation which occurred chiefly among the young and middle-aged generations because of fear of radiation exposure [14]. Minamisoma’s original population of 71,561 rapidly dropped to approximately 10,000 in the immediate aftermath of the disaster, and it has gradually recovered to 56,810 as of March 2017 [20]. Furthermore, the proportion of those aged 65 and above rose from 26.5% in 2010 to 32.0% in 2015, while the average number of people per household declined from 3.00 in 2010 to 2.23 in 2015 [21]. It has been additionally suggested that the average public expenditure for long-term care per person increased by 30% in the city post-disaster compared with pre-disaster because of changing demographics and the decreased social support and informal care available to the elderly remaining in the city [22]. However, there is still limited information available regarding health consequences induced by the post-disaster social changes in Minamisoma City.

Our case report describes the case of an elderly man diagnosed as having an advanced stage rectal cancer following long-term patient delay complicated by social isolation in Minamisoma City, Fukushima, after the triple disaster of March 2011 [14].

## Case presentation

In June 2016, an 80-year-old Japanese man with no significant medical history was referred to Minamisoma Municipal General Hospital (Fig. 1) for bloody stools and dizziness. He first noticed bloody stools in May 2015 and was aware of the possibility of colorectal cancer. However, he did not seek medical attention for over 1 year, reportedly because he considered the amount of blood trivial and did not experience other serious symptoms. It was only after considerable dizziness that he first sought medical consultation. His hemoglobin level was 7.4 g/dL with mean corpuscular volume of 78 fL at first presentation. Colonoscopy and abdominal computed tomography revealed a locally advanced rectal cancer.

He reported that he did not fear a cancer diagnosis. Regarding his life history, after he graduated from junior high school at age 15, he worked for the construction industry and retired at 73. Although he was married at 19 and had two children, he divorced at 42, and thereafter lived alone. He had never undertaken any type of cancer screening, although Minamisoma City provided multiple screening programs including biennial fecal occult blood tests for colorectal cancer [23]. He resided in the most central area of the city, with the nearest general hospital 1.0 km away.

Further detailed history taking revealed that he was exposed to social isolation after the 2011 disaster. Although he lived alone before the disaster, he hosted a neighborhood association with residents of the same generation and had many opportunities to socialize with friends. The association members were interested in health issues, and normally shared their health concerns with each other. In their gatherings, he had actually learned that colorectal cancer could cause bloody stools. However, the 2011 triple disaster, particularly the nuclear disaster, led to long-term evacuation of his neighbors. He lost contact with almost all his friends, and was unable to continue organizing the neighborhood association gatherings. He went outside less frequently and spent more time watching television, losing chances to discuss health concerns or share health information with others. In fact, he never talked to anyone about his symptoms before his first hospital visit. He remained out of contact with his ex-wife and children. Furthermore, he did not use a cellular phone, smart phone, or the Internet. He disclosed that he felt alone due to post-disaster changes, and that the diagnosis further intensified his loneliness, because he felt that he had “lost everything,” in terms of his family, friends, and his health. He also reported that he felt he should have made a more timely first medical consultation so that his disease might have been found at an earlier stage.

A Hartmann procedure was performed in June 2016 but it failed to successfully resect the lesion due to its severe invasion. The final diagnosis was stage IIIA rectal cancer with a direct invasion to sacrum. Although chemotherapy was initiated in July 2016, buttock pain which derived from the remnant lesion gradually exacerbated. We recommended palliative radiation therapy to him for the purpose of pain control, yet he decided not to receive it. Miyagi Cancer Center (Fig. 1), the most accessible facility with radiation equipment, is located approximately 60 km away from his residence; however, it was impossible for him to regularly visit the facility, as he did not possess a car and no one around him was able to take him there. He was admitted to our department due to general weakening in November 2016, and chemotherapy was stopped. We had multiple meetings with social workers and community health workers to try to achieve his wishes to spend his end-of-life period at home. However, he was not able to be discharged from the hospital due to insufficient support from his family and neighbors. He was transferred to a long-term care facility in January 2017, and died in February 2017.

## Discussion

This is a case of locally advanced rectal cancer with long-term patient delay following the 2011 triple disaster in Fukushima; our patient died within 1 year of his first medical consultation. We found that our patient experienced social isolation post-disaster, which may have reduced his opportunities to discuss health concerns and gain understanding of colorectal cancer; these factors may have contributed to the prolonged patient delay of over 1 year.

Social isolation is a crucial component which can complicate cancer management in post-disaster settings, as accentuated in Hurricane Katrina [24]. In fact, we previously reported an association between social isolation and patient delay in patients with breast cancer after the triple disaster [25], and the present case further underscores the possibility that face-to-face interaction with family and friends can play a predominant role as a source for health information, and as an opportunity to discuss and confirm symptoms. In addition, socially isolated elderly adults may find it particularly difficult to adopt new sources of health information, such as the Internet [9]. We therefore find it important to recognize that events triggering social isolation, such as nuclear disasters, may have widespread health consequences through loss of health information and social support, particularly in elderly populations.

Of note, although our patient was initially aware of the potential of malignancy, he did not pursue this possibility. Normalcy bias, a mindset which underestimates possible risk and assumes that adverse consequences occur to other people, may have been a potential contributor to his actions [25, 26]. Social isolation may strengthen normalcy bias [25], and if he had had an opportunity to discuss his symptoms with family members or friends, he may have been able to interpret his own symptoms appropriately and seek medical attention earlier; these processes have been found to be crucial for seeking timely medical care [27]. Furthermore, his limited health literacy and initial lack of urgent symptoms call for further attention in fully understanding his patient delay, as they are known risk factors [8]. We speculate that the coexistence of the above factors with social isolation could have made him more susceptible to patient delay.

He reported a continued intense feeling of loneliness for all the 5 years following the disaster. Social isolation may be more persistent after nuclear disasters, compared to natural disasters, due to long-lasting evacuation and community tensions [25, 28]. Community disruption after the nuclear accident appears to have caused social isolation in our patient, and other patients with cancer in Fukushima may be experiencing similar phenomena of isolation-induced delay, given that the restoration from the Fukushima nuclear disasters is still underway 6 years after the disaster [29, 30].

The social isolation of our patient not only led to his patient delay and an advanced stage diagnosis, but also limited his treatment choices and eventually lead to an early death. He was able to receive neither radiation therapy nor home-based care in his end-of-life period. These findings suggest that conventional support from health care providers and social workers alone may not be enough to overcome social isolation. It is true that the World Health Organization and the United Nations Scientific Committee on the Effects of Atomic Radiation (UNSCEAR) have concluded that any possible health effects induced by radiation exposure after the Fukushima disaster would be negligible [31, 32], and this information, along with new results from ongoing studies, is shared widely with local residents in Fukushima through public presentations and media coverage. However, health issues caused by social changes following the 2011 triple disaster, as accentuated in this case, have been relatively underrepresented.

Local health care providers have been playing extra roles to alleviate the long-term decline of social support in the disaster-struck areas of Fukushima [29]. Although the provision of medical services is the most critical task for physicians, and individual social workers and community health workers may be able to intervene in patients’ social environments through public and private services, these hospital-centered measures alone are unlikely to resolve delayed medical consultations among socially isolated people [25]. Although it is difficult to individually reach residents with cancer-suggestive symptoms who are yet to make their first medical consultation, it may be worthwhile to organize public sessions which promote cancer awareness and knowledge among local residents, possibly enabling local health care providers to approach a large number of patients with potential cancer symptoms.

We additionally suggest that policy-level interventions are imperative in resolving social isolation and its persistent health effects following the Fukushima nuclear disaster. In particular, it may be meaningful for local municipalities to conduct community outreach programs, such as health checkups and counseling services, at shelters and temporary housing sites in addition to general residential areas [33–35]. These efforts may help to identify local residents with insufficient support or those with early signs of cancer, and encourage these vulnerable populations to make timely medical consultations when they are in need of medical services. Considering that isolation-induced health effects are not limited to patients with cancer [36], mitigation of isolation should be considered a critical public health issue in post-disaster settings. Public activities to strengthen the bonds and interactions within communities may further alleviate social isolation and its broad health effects.

Given recent global trends of population ageing, nuclear power use, and climate change, the present findings may be applicable beyond Fukushima. Population ageing has progressed globally, with longevity and declining fertility rates [37]. In particular, low fertility rates, an issue among the majority of HICs [37], can contribute to social isolation in the elderly, as accentuated in examples from China [38]. In addition, while there is increasing interest in nuclear energy [39], concerns remain about future nuclear disasters [40]. Natural disasters may similarly expose their victims to social isolation and its various health consequences, as seen in reports from Hurricane Katrina [41, 42], and the occurrence and severity of this type of disaster are currently on the rise worldwide due to global warming [43]. The potential for a simultaneous increase of elderly populations and risk of both nuclear and natural disasters calls for improved attention to the broader impacts of social isolation among the elderly.

## Conclusions

Social isolation after the nuclear accident may have been a primary contributing factor to patient delay in this case. Social support from family and friends may be imperative for the health of the elderly, contributing to their health awareness and health-seeking behaviors; social isolation can therefore delay a first medical consultation and diagnosis, in addition to the course of treatment and outcome. Countermeasures led by health care providers and broader policy initiatives may be warranted to reduce social isolation and its health effects. This case additionally suggests we should be aware that disasters can further deteriorate social connections of elderly populations, possibly leading to a detrimental effect on post-disaster management of health issues, including cancers.

## Abbreviations

FDNPP: Fukushima Daiichi Nuclear Power Plant
HICs: High-income countries
OECD: Organisation for Economic Co-operation and Development
UNSCEAR: United Nations Scientific Committee on the Effects of Atomic Radiation
